# Chronic kidney disease promotes cerebral microhemorrhage formation

**DOI:** 10.1186/s12974-023-02703-2

**Published:** 2023-02-25

**Authors:** Chuo Fang, Wei Ling Lau, Jiahong Sun, Rudy Chang, Adrian Vallejo, Donghy Lee, Jihua Liu, Han Liu, Yu-Han Hung, Yitong Zhao, Annlia Paganini-Hill, Rachita K. Sumbria, David H. Cribbs, Mark Fisher

**Affiliations:** 1 Department of Neurology, University of California, Irvine, CA USA; 2 Department of Medicine, Division of Nephrology, University of California, Irvine, CA USA; 3 Department of Biomedical and Pharmaceutical Sciences, School of Pharmacy, Chapman University, Irvine, CA USA; 4 Institute for Memory Impairments and Neurological Disorders, University of California, Irvine, CA USA; 5 Department of Pathology & Laboratory Medicine, University of California, Irvine, CA USA; 6 Department of Neurology, UC Irvine Medical Center, 101 The City Drive South, Shanbrom Hall (Building 55), Room 121, Orange, CA 92868 USA

**Keywords:** Cerebral microhemorrhages/microbleeds, Aging, Chronic kidney disease, Microglial activation, Brain endothelial cells, Blood–brain barrier, Uremic toxins

## Abstract

**Background:**

Chronic kidney disease (CKD) is increasingly recognized as a stroke risk factor, but its exact relationship with cerebrovascular disease is not well-understood. We investigated the development of cerebral small vessel disease using in vivo and in vitro models of CKD.

**Methods:**

CKD was produced in aged C57BL/6J mice using an adenine-induced tubulointerstitial nephritis model. We analyzed brain histology using Prussian blue staining to examine formation of cerebral microhemorrhage (CMH), the hemorrhagic component of small vessel disease and the neuropathological substrate of MRI-demonstrable cerebral microbleeds. In cell culture studies, we examined effects of serum from healthy or CKD patients and gut-derived uremic toxins on brain microvascular endothelial barrier.

**Results:**

CKD was induced in aged C57BL/6J mice with significant increases in both serum creatinine and cystatin C levels (*p* < 0.0001) without elevation of systolic or diastolic blood pressure. CMH was significantly increased and positively correlated with serum creatinine level (Spearman *r* = 0.37, *p* < 0.01). Moreover, CKD significantly increased Iba-1-positive immunoreactivity by 51% (*p* < 0.001), induced a phenotypic switch from resting to activated microglia, and enhanced fibrinogen extravasation across the blood–brain barrier (BBB) by 34% (*p* < 0.05). On analysis stratified by sex, the increase in CMH number was more pronounced in male mice and this correlated with greater creatinine elevation in male compared with female mice. Microglial depletion with PLX3397 diet significantly decreased CMH formation in CKD mice without affecting serum creatinine levels. Incubation of CKD serum significantly reduced transendothelial electrical resistance (TEER) (*p* < 0.01) and increased sodium fluorescein permeability (*p* < 0.05) across the endothelial monolayer. Uremic toxins (i.e., indoxyl sulfate, p-cresyl sulfate, and trimethylamine-N-oxide) in combination with urea and lipopolysaccharide induced a marked drop in TEER compared with the control group (*p* < 0.0001).

**Conclusions:**

CKD promotes the development of CMH in aged mice independent of blood pressure but directly proportional to the degree of renal impairment. These effects of CKD are likely mediated in part by microglia and are associated with BBB impairment. The latter is likely related to gut-derived bacteria-dependent toxins classically associated with CKD. Overall, these findings demonstrate an important role of CKD in the development of cerebral small vessel disease.

## Background

Chronic kidney disease (CKD) is a major public health issue that affects 15% of U.S. adults, leading to impaired quality of life [1]. Meta-analysis and systemic review of population-based studies provide strong evidence supporting CKD as an independent risk factor for cerebral small vessel disease and cognitive impairment [2–4]. Notably, end-stage renal disease is associated with a substantially higher risk of both ischemic and hemorrhagic strokes [3] as well as accelerated brain aging and cognitive decline [5], and consequently, higher morbidity and mortality [6]. Postmortem examination of CKD human brains showed an increased prevalence of cerebral small vessel disease and highlighted the frequency of microvascular calcification in those brains [7]. Pathways that promote cerebral small vessel disease in the uremic milieu include loss of calcium/phosphorus homeostasis, blood pressure variability, retention of vascular toxins, and chronic inflammation [8]. Investigations into the relationship between CKD and cerebral small vessel disease subtypes are needed to identify novel prevention and treatment strategies in CKD patients.

Cerebral microhemorrhages (CMH) are the pathological substrate for cerebral microbleeds, which represent focal hemosiderin/iron deposits on MRI and are linked to cognitive impairment and ischemic and hemorrhagic stroke. Given that cerebral microbleeds are strongly age-dependent [9] and are present in up to 50% of hemodialysis patients [10], microbleeds may predict vascular outcomes in CKD patients [11, 12]. However, the relationship between CKD and development of cerebral microbleeds is not well-understood in human studies due to concurrent comorbidities such as diabetes and hypertension that also affect the cerebral microvasculature [7]. In comparison, animal models have the advantage of isolating the effect of CKD from other vascular risk factors and thus can provide insights into the direct relationship between CKD and CMH. We previously described a mouse CKD model with adenine-induced tubulointerstitial nephritis and reported more than two-fold increase in CMH burden during an inflammatory state [13]. We previously showed that aging increased inflammation-induced CMH, likely by microglial activation and blood–brain barrier (BBB) disruption [14]. CKD promotes premature aging by inducing inflammation and oxidative stress via a wide range of risk factors including uremic toxins, dialysis, epigenetic factors, and cellular senescence [15]. However, whether CKD mediates age-related CMH formation and underlying pathogenic mechanisms have remained unclear.

Gut-derived uremic toxins, notably endotoxin (lipopolysaccharide, LPS), indoxyl sulfate (IS), p-cresyl sulfate (PCS), and trimethylamine-N-oxide (TMAO) [16–18], along with urea [13, 13] can exert deleterious effects on brain microcirculation, neurons, microglia, and astrocytes [19]. Beyond that, uremic toxins can exacerbate systemic and local inflammation [19] that may further enhance the pre-existing CMH burden [14]. Our previous work has shown that uremic toxins in CKD patients reduced transendothelial electrical resistance (TEER) and tight junction proteins of a murine brain endothelial cell monolayer in a dose-dependent manner [13], supporting a mechanistic role of uremic toxins in BBB disruption and the development of CMH. We therefore performed the current study to further investigate the relationship between CKD and CMH development in a mouse model of aging. We also examined the effect of CKD serum and uremic toxins on brain microvascular endothelial cell function in vitro*.* Our results help expand knowledge of brain microvascular consequences of CKD and provide mechanistic insights.

## Materials and methods

### In vivo experiments

#### Animals and chronic kidney disease model

**Fig. 1:**
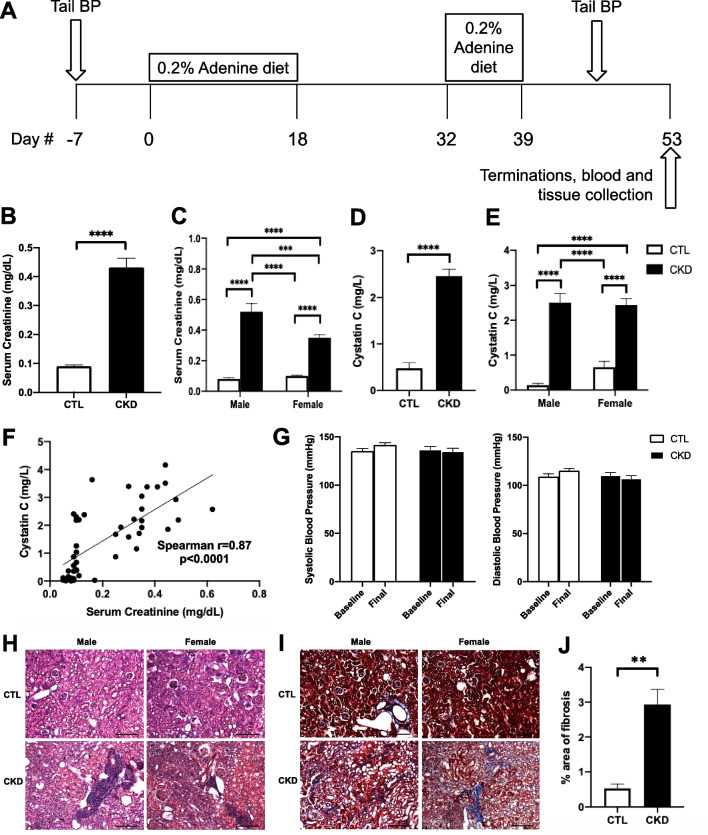
Adenine-induced chronic kidney disease (CKD) mouse model. **A** Experimental timeline. Aged (17 months) C57BL/6J mice were placed on a diet containing 0.2% adenine for 18 days, followed by 2 weeks of regular diet, and then re-exposed to 0.2% adenine-containing diet for 1 week to maintain CKD induction. Mice were terminated 2 weeks after the second adenine exposure was completed. **B**, **C** Mice with adenine-induced CKD showed significantly higher serum creatinine levels (mg/dL) in both males and females compared with control (CTL) mice. **D**, **E** Serum cystatin C (mg/L) was elevated in CKD animals and **F** correlated closely with serum creatinine. **G** Systolic and diastolic blood pressure (mmHg) were assessed prior to initial CKD induction (Baseline) and termination (Final) using the non-invasive tail-cuff technique. **H** Representative photomicrographs of kidney H&E from CTL and CKD mice. CKD animals demonstrated interstitial inflammation and tubular injury. **I** Representative photomicrographs of kidney Masson’s trichrome staining from CTL and CKD animals. CTL mice demonstrated some collagen deposition (blue) around arterioles (normal finding). **J** CKD animals had increased parenchymal fibrosis (blue) consistent with chronic injury. Scale bar = 200 μm. Data shown are mean ± SEM. *n* = 15–17 per sex per group. **p < 0.01, ***p < 0.001 and ****p < 0.0001

Aged (17 months) C57BL/6J female and male mice (National Institute of Aging, Bethesda, MD) were randomly divided into control and CKD groups (*n* = 15–17 per sex per group). Progressive kidney injury was induced in 17-month-old mice using an adenine-induced tubulointerstitial nephritis model, which is equivalent to CKD development over 4 years in 55-year-old humans [20]. After 1 week of acclimation, mice were placed on a diet containing 0.2% adenine (Sigma-Aldrich, St. Louis, MO, USA; diet manufactured at Dyets Inc., Bethlehem, PA) for 18 days, followed by regular chow for 2 weeks, and then re-exposed to adenine diet for 1 week to maintain CKD. Mice were terminated 2 weeks after the second adenine exposure (Fig. 1A) [13]. Systolic and diastolic blood pressure levels were assessed prior to initial CKD induction (Baseline) and termination (Final) using the non-invasive tail-cuff technique (CODA, Kent Scientific, Torrington, CT, USA) [21]. All experimental procedures followed the NIH Guide for the Care and Use of Laboratory Animals and were approved by the Institutional Animal Care and Use Committee at the University of California, Irvine.

#### Microglial depletion model

To investigate the role of microglia in CKD-induced CMH formation, we used a highly selective colony-stimulating factor 1 receptor (CSF1R) inhibitor PLX3397 (pexidartinib, MedChemExpress, New Jersey, USA), which reduces microglia number by up to 99% after 3 weeks of treatment [22]. As shown in Fig. 4A, CKD was induced via 0.2% adenine diet in aged (17 months) C57BL/6J female and male mice as described above. Following the second adenine exposure, mice received regular chow or PLX3397 diet (290 mg/kg, diet manufactured at Dyets Inc., Bethlehem, PA) for 3 weeks prior to mouse terminations. Systolic and diastolic blood pressure levels were assessed as described above.

#### Tissue collection and blood chemistries

Mice were euthanized with inhaled isoflurane followed by cardiac perfusion with ice-cold phosphate-buffered saline (PBS) for 5 min. Brains were harvested, fixed overnight in 4% paraformaldehyde (Thermo Fisher Scientific, Cleveland, OH, USA), and passaged through 15% and 30% sucrose solutions prior to storage at − 70 °C. Brains were then processed for histological analyses as described previously [14]. Serum samples were collected for creatinine measurements via isotope dilution liquid chromatography with tandem mass spectrometry (LC–MS/MS) at the O’Brien Center Bioanalytical Core (University of Alabama at Birmingham, Birmingham, AL, USA). Serum cystatin C was measured using the mouse ELISA kit (Invitrogen, Carlsbad, CA, USA) per manufacturer’s protocol.

#### Kidney histopathological analysis

Ten-micron kidney sections were generated using a freezing microtome, stained with hematoxylin and eosin (H&E) or Masson’s trichrome, and examined under a photomicroscope (Nikon Eclipse, Japan). An ImageJ macro was used to quantify kidney fibrosis (% area stained blue relative to red on Masson’s trichrome) as previously described [23, 24]. Six images of kidney cortex were captured at 10× magnification from 3 animals per group by an observer blinded to the study groups, and mean % area was calculated per animal.

#### Detection and quantification of CMH

The brains were sectioned into 20-μm or 40-µm coronal sections with a freezing microtome (Thermo Fisher Scientific, Cleveland, OH, USA). Every seventh section was collected for Prussian blue staining to detect hemosiderin (marker of CMH formation) performed by the Research Services Core (Department of Pathology and Laboratory Medicine at UCI Medical Center). Briefly, sections were stained with freshly prepared 5% potassium hexacyanoferrate trihydrate (Sigma-Aldrich, St. Louis, MO, USA) and 10% hydrochloric acid (Sigma-Aldrich, St. Louis, MO, USA) for 30 min. After rinsing in water, sections were counterstained with nuclear fast red, dehydrated, and coverslipped. To quantify CMH number, CMH were detected and photographed at 20× magnification with a light microscope by a blinded observer. Whole slide images were scanned to quantify the total area of the brain section. The area of the section was analyzed by a blinded observer using National Institute of Health (NIH) ImageJ software 1.52. CMH number was then adjusted to total area of the brain section [13].

#### Immunohistochemistry for neuroinflammation and BBB disruption

To detect neuroinflammation and BBB disruption, immunohistochemistry for Iba-1 (microglia/macrophages marker), glial fibrillary acidic protein (GFAP, astrocyte marker), and fibrinogen (BBB injury marker) was performed. Briefly, 20-μm or 40-µm coronal sections were incubated in 0.3% hydrogen peroxide in 1 × PBS (pH 7.4) for 30 min at room temperature (RT), blocked with PBST containing 1% bovine serum albumin for 2 h at RT. Sections were then incubated overnight at 4° C with rabbit primary antibodies against Iba-1 (1:400 dilution, Wako Chemicals USA, Richmond, VA, USA), GFAP (1:2000 dilution, Abcam, Cambridge, MA, USA), or fibrinogen (1:50 dilution, Abcam, Cambridge, MA, USA). After washing with PBST, sections were incubated with biotinylated anti-rabbit secondary IgG (1:1000 dilution, Jackson ImmunoResearch, West Grove, PA, USA) for 2 h at RT. After washing with PBS, sections were incubated with avidin–biotin-peroxidase (ABC) complex (Vector Laboratories, Burlingame, CA, USA) for 45 min at RT and developed using 3,3′-diaminobenzidine (DAB) (Vector Laboratories, Burlingame, CA, USA). Thirteen images per brain section were acquired from three brain regions (cortex, hippocampus, and thalamus) at 20× magnification. Quantification of immunoreactive area (expressed as percentage of total analyzed area) was performed by a blinded observer using NIH ImageJ software 1.52, and the average immunoreactive area of three brain regions was calculated [13]. To determine the morphological state of microglia, Iba-1-stained sections were photographed at 40× magnification, and microglia were categorized into the resting or activated state by a blinded observer. Resting microglia are characterized by long, ramified processes with small cell bodies. In disease states, microglia can be activated and transformed into a phagocytic appearance, exhibiting large, rounded cell bodies with no or few processes [25]. The number of microglia in each state was expressed as the percentage of total microglia.

### In vitro experiments

#### Human induced pluripotent stem cell (iPSC) culture and differentiation

iPSC IMR90 clone 4 line (IMR90-4, WiCell, Madison, WI, USA, passage 34–37; Catalog no. WIS-Ci004-B) were differentiated into human brain microvascular endothelial cells (ihBMEC) as described previously [26]. Briefly, IMR90-4 cells were maintained in mTeSR™-Plus medium (STEMCELL Technologies, Vancouver, BC, Canada). For differentiation, IMR90-4 cells were detached by Accutase (Innovative Cell Technologies, San Diego, CA, USA) and re-plated onto Matrigel-coated 6-well plates at a density of 2.5 × 10^4^ cells/cm^2^ in mTeSR™-Plus medium supplemented with 10 μM rho-associated protein kinase (ROCK) inhibitor Y-27632 (Selleckchem, Houston, TX, USA) for 3 days. Cell differentiation was induced by treating IMR90-4 cells with Dulbecco's Modified Eagle Medium/Ham’s nutrient mixture F-12 (DMEM/Ham’s F12) medium (Thermo Fisher, Waltham, MA, USA) supplemented with 6 μM glycogen synthase kinase 3 inhibitor/Wnt activator CHIR99021 (Selleckchem, Houston, TX, USA), 1× minimum essential medium–nonessential amino acids (MEM-NEAA) (Thermo Fisher, Waltham, MA, USA), 0.5× GlutaMAX™ (Thermo Fisher, Waltham, MA, USA), and 0.1 mM β-mercaptoethanol (Sigma-Aldrich, St. Louis, MO, USA) for 1 day. To maintain differentiation, medium was replaced with DMEM/Ham’s F12 medium supplemented with 1X MEM-NEAA, 0.5× GlutaMAX™, and 0.1 mM β-mercaptoethanol every 24 h. After 5 days, medium was replaced with human endothelial serum-free medium (hESFM, Invitrogen, Carlsbad, CA, USA) supplemented with 20 ng/mL basic fibroblast growth factor (bFGF), 1× B27 supplement (Thermo Fisher, Waltham, MA, USA) and 10 µM retinoic acid (Sigma-Aldrich, St. Louis, MO, USA) for 3 days for complete differentiation to ihBMEC. Each batch of differentiated ihBMEC was examined for their endothelial phenotype by confirming their cobblestone morphology, expression of endothelial marker von Willebrand factor (vWF), and uptake of acetylated low-density lipoprotein (LDL), as described previously [26].

#### Murine brain microvascular endothelial cell culture

bEnd.3 cells (American Type Culture Collection, Manassas, VA, USA; Catalog no. CRL-2299) were cultured in Dulbecco’s Modified Eagle’s Medium (DMEM, American Type Culture Collection, Manassas, VA, USA) supplemented with 10% fetal bovine serum (FBS, R&D systems, Minneapolis, MN, USA), and 100 μg/mL penicillin/streptomycin (Sigma-Aldrich, St. Louis, MO, USA) at 37 °C (5% CO_2_, 95% air). The endothelial phenotype of the bEnd.3 cells was confirmed by evaluating their cobblestone morphology, expression of endothelial marker vWF, and uptake of acetylated LDL, as described previously [26]. Cells between passage numbers 25–35 were seeded at a density of 1 × 10^5^ to 1 × 10^6^ cells/cm^2^ and grown to confluence on 24-well Transwell inserts (pore size 0.4 μm, Corning Inc., Corning, NY, USA) [26]. Cells were then exposed to uremic toxins for 4 days prior to further experiments. Urea 72 mg/dL, LPS 10 ng/mL, mixture of gut-derived uremic toxins (3Toxins = IS 150 µg/mL + PCS 100 µg/mL + TMAO 1 mg/mL) were tested alone and in combination. The reference group was cells grown in DMEM with 10% FBS, and mannitol 450 mg/dL was used for an osmotic control group.

#### TEER measurement

ihBMEC were replated at a density of 1 × 10^6^ cells/cm^2^ on 24-well Transwell inserts (pore size 0.4 μm, Corning Inc., Corning, NY, USA) coated with a solution containing 400 μg/mL collagen IV (Sigma-Aldrich, St. Louis, MO, USA) and 100 μg/mL fibronectin (Sigma-Aldrich, St. Louis, MO, USA). To examine the effect of CKD on BBB integrity, ihBMEC were treated with human healthy serum or uremic serum from dialysis patients for 3 days. Based on our prior work in a mouse brain endothelial BBB model in which incubation with CKD serum produced a dose-dependent drop in TEER [13], 15% serum concentration was chosen. Three days of incubation was chosen, because the trophic factors in human serum are sufficient to maintain cell growth and health within 3 days [26]. In a separate experiment, effects of uremic toxins on the bEnd.3 endothelial barrier were assessed by exposing bEnd.3 cells to uremic toxins for 4 days. Integrity of the endothelial monolayer was assessed by measuring TEER using the EVOM2 Epithelial Volt/Ohm Meter and an STX-2 electrode system (World Precision Instruments LLC, Sarasota, FL, USA) every 24 h. TEER values were calculated by subtracting the resistance of cell-free blank Transwells [26]. TEER values for each treatment were expressed as the percentage TEER of the baseline values. Human serum samples from healthy individuals and from end-stage renal disease patients on chronic hemodialysis were obtained from previously banked samples after IRB approval and informed consent.

#### Tracer permeability measurement

Tracer permeability across the ihBMEC cell monolayer was assessed after TEER values were stabilized. Sodium fluorescein (5 μg/mL, MW: 376 Da, Sigma-Aldrich, St. Louis, MO, USA) was added to the medium in the apical chamber, and medium in the basolateral chamber was collected after 3 days to determine the passage of sodium fluorescein. The mean fluorescent intensity of sodium fluorescein was measured using a fluorescence plate reader (Molecular Devices, LLC, San Jose, CA, USA) at an excitation/emission wavelength of 460 nm/515 nm. Tracer permeability was expressed as permeability coefficient (cm/s) calculated as described previously [26].

#### Cell viability assay

ihBMEC (7500 cells/well) were seeded in 96-well plates (Corning, New York, NY, USA). Cell viability was measured using the Cell Counting Kit-8 (CCK-8) assay according to manufacturer’s instructions (Dojindo Molecular Technologies, Rockville, MD, USA) 3 days after serum treatment initiation. Absorbance was measured at 450 nm using a plate reader (Molecular Devices, LLC, San Jose, CA, USA). Cell viability was expressed as the percentage of ihBMEC controls without serum treatment [26]. bEnd.3 cells (3 × 10^5^ cells/well) were seeded in 6-well plates (Corning, New York, NY, USA) and treated with uremic toxins. Cell viability was measured using trypan blue assay according to manufacturer’s instructions (Sigma-Aldrich, St. Louis, MO, USA) 3 days after treatment initiation. Cell viability was expressed as the percentage of total viable cells (unstained) in total cells (stained and unstained).

### Statistical analysis

The data were analyzed by GraphPad Prism 8 (GraphPad Software, Lo Jolla, CA) and SAS version 9.4 (SAS Institute Inc., Cary, NC). Results are presented as means ± SEM. Normality tests were performed using D’Agostino-Pearson. Comparison of means was analyzed by Student’s *t* test for two independent groups, by two-way ANOVA (e.g., factor 1 treatment CTL/CKD and factor 2 male/female) with post-hoc Sidak-adjusted pairwise comparisons, or by two-way repeated measures ANOVA for timepoint and treatment with post-hoc comparisons. To determine the association between variables, Spearman’s correlation tests were performed, and results were presented as correlation coefficient (r). P value of less than 0.05 was considered statistically significant.

## Results

### Adenine-induced CKD promoted CMH formation in aged C57BL/6 J mice without altering blood pressure levels

In this adenine-induced CKD mouse model, serum levels of creatinine, a biomarker of kidney insufficiency, were increased 3.8-fold in CKD mice (0.43 ± 0.03 mg/dL vs. 0.09 ± 0.01 mg/dL in controls, *p* < 0.0001) (Fig. 1B). Serum creatinine values were significantly higher in CKD males than CKD females (0.52 ± 0.05 mg/dL vs. 0.35 ± 0.02 mg/dL, *p* < 0.001) (Fig. 1C). Another marker of kidney injury, cystatin C, was similarly elevated in CKD animals and correlated closely with serum creatinine (Spearman *r* = 0.87, *p* < 0.0001) (Fig. 1D–F). Average serum cystatin C was 0.14 ± 0.05 mg/L and 0.66 ± 0.16 mg/L in control male and female animals; and was 2.5 ± 0.26 mg/L and 2.44 ± 0.18 mg/L in CKD male and female animals (Fig. 1E). H&E staining of kidney sections demonstrated interstitial inflammation and tubular injury in CKD animals (Fig. 1H). On kidney trichrome staining, area of fibrosis was increased 5.5-fold (p < 0.01) in CKD animals compared with controls (0.5 ± 0.1 vs. 2.9 ± 0.4% area of fibrosis, respectively) (Fig. 1I, and J). There was no difference in fibrosis scores between the male and female kidneys from CKD animals (data not shown). This adenine-induced CKD model is non-hypertensive; pre- and post-CKD systolic (137 ± 3 mmHg to 135 ± 3 mmHg) and diastolic blood pressure (110 ± 3 mmHg to 107 ± 3 mmHg) remained essentially unchanged (Fig. 1G).

**Fig. 2:**
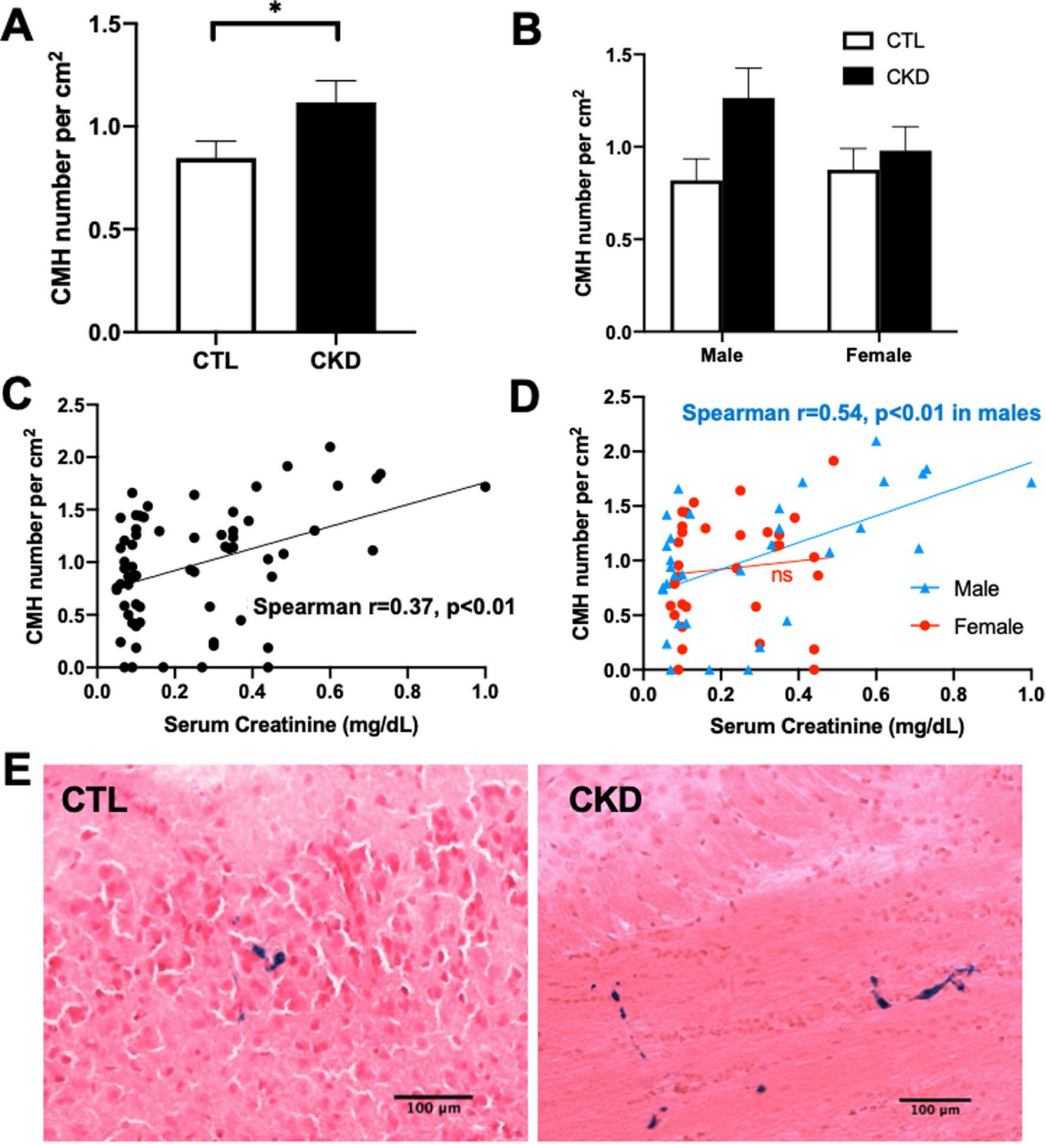
CKD induction promoted cerebral microhemorrhages (CMH) formation. **A**, **B** Significantly higher CMH number, expressed as Prussian blue-positive CMH, in CKD mice compared with CTL mice. **C**, **D** Prussian blue positivity was positively correlated (Spearman correlation) with serum creatinine values. **E** Representative images of Prussian blue-positive lesions in the subcortex from CTL and CKD mice, indicating CMH burden. Scale bar = 100 μm. Data shown are mean ± SEM. *n* = 15–17 per sex per group. *p < 0.05

CMH burden was measured by the number of Prussian blue-positive CMH adjusted to total area of the brain section (CMH number per cm^2^) (Fig. 2A–E). Prussian blue staining revealed that the average CMH number was significantly higher in CKD mice compared with control mice (1.12 ± 0.10 per cm^2^ vs. 0.85 ± 0.08 per cm^2^, p < 0.05) (Fig. 2A, and E). CKD-induced increase in CMH number was more pronounced in males (1.26 ± 0.16 per cm^2^ vs. 0.82 ± 0.12 per cm^2^ in control males, *p* = 0.11) than in females (0.98 ± 0.13 per cm^2^ vs. 0.88 ± 0.11 per cm^2^ in control females, *p* = 0.99) (Fig. 2B). CMH number was positively correlated with serum creatinine values in aged mice (Spearman *r* = 0.37, *p* < 0.01, Fig. 2C). This positive association was notably greater in males (Spearman *r* = 0.54, *p* < 0.01, Fig. 2D), which is in line with a larger increase in serum creatinine in CKD males compared with CKD females. Therefore, CMH burden was significantly increased and associated with the degree of adenine-induced kidney injury.

### CKD induction promoted microglial activation and BBB disruption

**Fig. 3:**
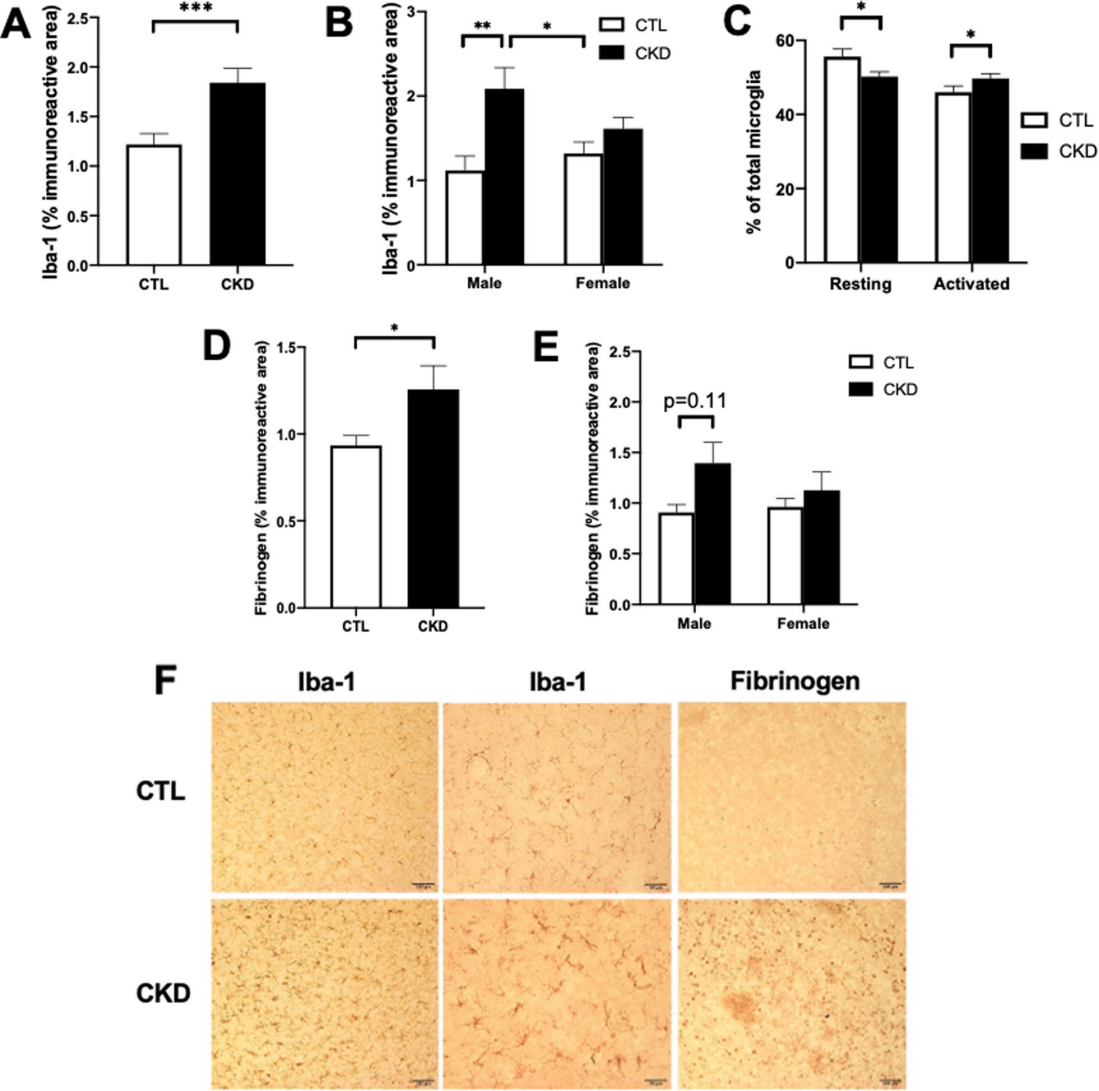
CKD induction promoted microglial activation and blood–brain barrier (BBB) disruption. **A**, **B** Significantly higher Iba-1 immunoreactivity, expressed as % of Iba-1 immunoreactive area, in CKD mice compared with CTL mice. **C** Number of microglia within the two states (resting and activated) expressed as a proportion of the total number of microglia was quantified. CKD mice showed decreased number of resting microglia and increased number of activated microglia compared with CTL mice. **D**, **E** Fibrinogen immunoreactivity, expressed as % of immunoreactive area, was significantly higher in CKD mice compared with CTL mice. **F** Representative immunohistochemical images of Iba-1 (left: 20× objective, scale bar = 100 μm; middle: 40× objective, scale bar = 50 μm) and fibrinogen (right: 20× objective, scale bar = 100 μm) in the thalamus. Data shown are mean ± SEM. *n* = 15–17 per sex per group. *p < 0.05, **p < 0.01 and ***p < 0.001

To determine if adenine-induced CKD is associated with glial activation and BBB disruption, we performed immunohistochemistry for Iba-1, GFAP, and fibrinogen (Fig. 3A–F). The number and morphology of microglia were quantified and expressed as the percentage of Iba-1 immunoreactive area and percentage of the number of resting and activated microglia as compared to total microglia, respectively. After CKD induction, Iba-1-positive immunoreactivity increased by 51% compared with controls (1.84 ± 0.14 vs. 1.22 ± 0.11% of analyzed brain area, *p* < 0.001) (Fig. 3A, and F), which was accompanied by a small increase in the proportion of activated microglia (49.7% ± 1.3 vs. 45.4% ± 1.5, *p* < 0.05) and a corresponding significant decrease in resting microglia (data not shown) (Fig. 3C). GFAP staining was not significantly different between CKD and control mice (4.31 ± 0.62 vs. 3.46 ± 0.3%). Fibrinogen-positive immunoreactivity was significantly higher by 34% in CKD mice compared with CTL mice (1.26 ± 0.14 vs. 0.93 ± 0.06, *p* < 0.05) (Fig. 3D, and F). CKD significantly increased Iba-1 immunoreactivity in males compared with females (p < 0.01, Fig. 3B); however, no sex differences were seen with fibrinogen (Fig. 3E). Overall, CKD induction promoted microglial activation and BBB disruption in aged mice, with changes being more pronounced in CKD males than CKD females.

### Microglial depletion with PLX3397 diet significantly decreased CMH in CKD mice

**Fig. 4:**
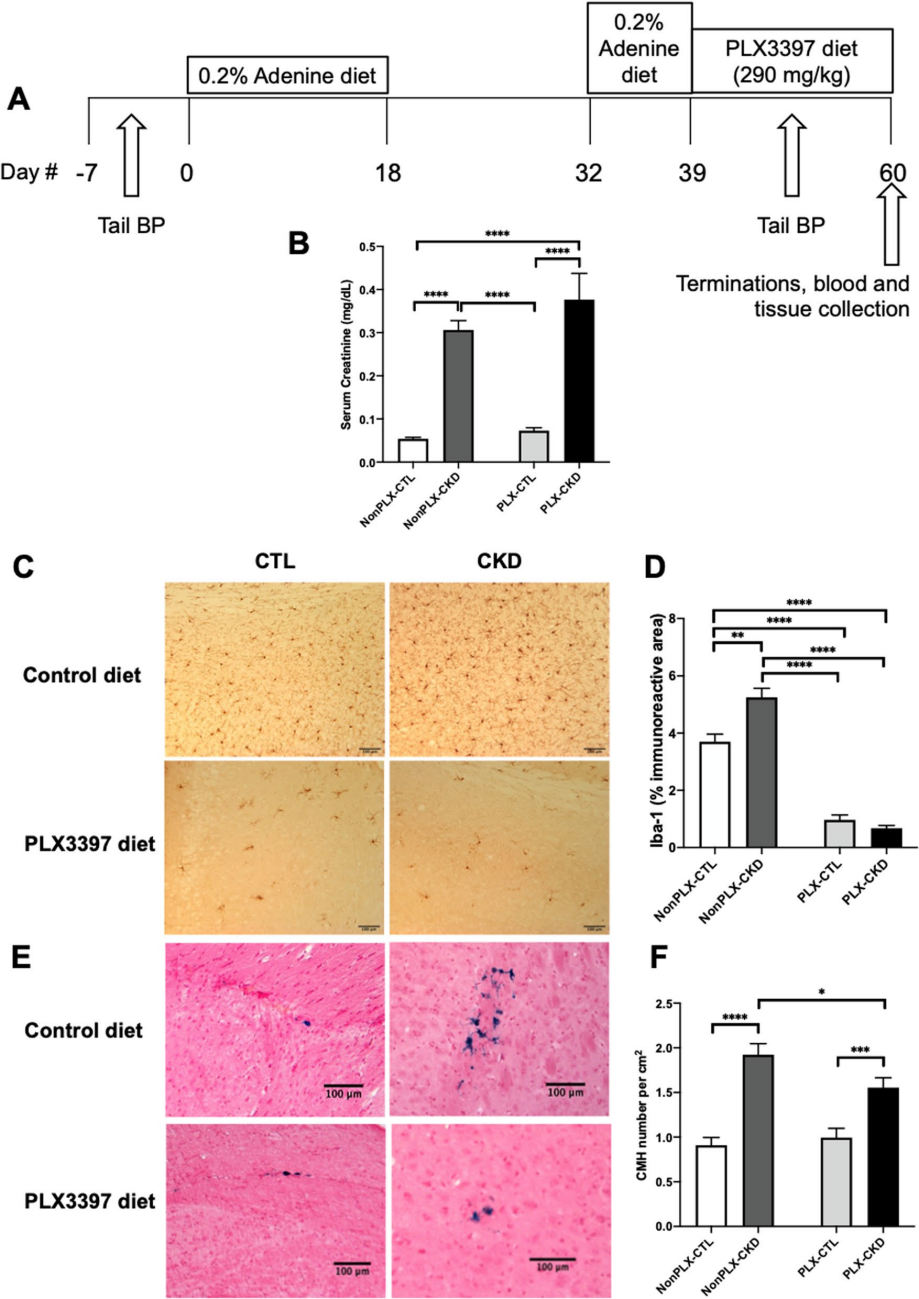
Microglial depletion with PLX3397 diet significantly decreased CMH in CKD mice. **A** Experimental timeline. To achieve microglial depletion in CKD mice, aged (17 months) C57BL/6J mice were placed on PLX3397 diet (PLX, 290 mg/kg in regular chow) for 3 weeks after completion of the second adenine exposure. **B** Mice with adenine-induced CKD showed significantly higher serum creatinine levels (mg/dL) compared with CTL mice regardless of microglial depletion. **C** Representative images of Iba-1 immunohistochemical staining in the cortex from CTL and CKD mice with or without microglial depletion, showing **D** Iba-1 immunoreactive area was significantly lower in CTL and CKD mice after microglial depletion. **E** Representative images of Prussian blue-positive lesions in the subcortex from CTL and CKD mice with or without microglial depletion, showing **F** CMH formation in CKD mice was significantly decreased after microglial depletion. Scale bar = 100 μm. Data shown are mean ± SEM. *n* = 10 in NonPLX–CTL group, 10 in PLX–CTL group, 36 in NonPLX–CKD group, and 25 in PLX–CKD group. *p < 0.05, **p < 0.01, ***p < 0.001 and ****p < 0.0001

We examined the mechanistic link between microglial activation and CMH formation in CKD using PLX3397 diet, which allows for microglial depletion during CMH formation post-CKD induction. As expected, mice with adenine-induced CKD showed significantly higher serum creatinine levels (Fig. 4B) and CMH number compared with control mice (Fig. 4E, and F). Immunostaining for Iba-1showed marked decrease in Iba-1 immunoreactive area (*p* < 0.0001) in the mouse brains after 3 weeks of PLX3397 diet (Fig. 4C, and D). Microglial depletion with PLX3397 significantly reduced CMH formation in CKD mice from 1.92 ± 0.12 per cm^2^ to 1.55 ± 0.11 per cm^2^ (*p* < 0.05) (Fig. 4E, and F). No significant differences were observed for systolic and diastolic blood pressure with and without the PLX3397 diet (data not shown).

### Serum-derived factors in CKD disrupted BBB integrity of the ihBMEC monolayer

**Fig. 5:**
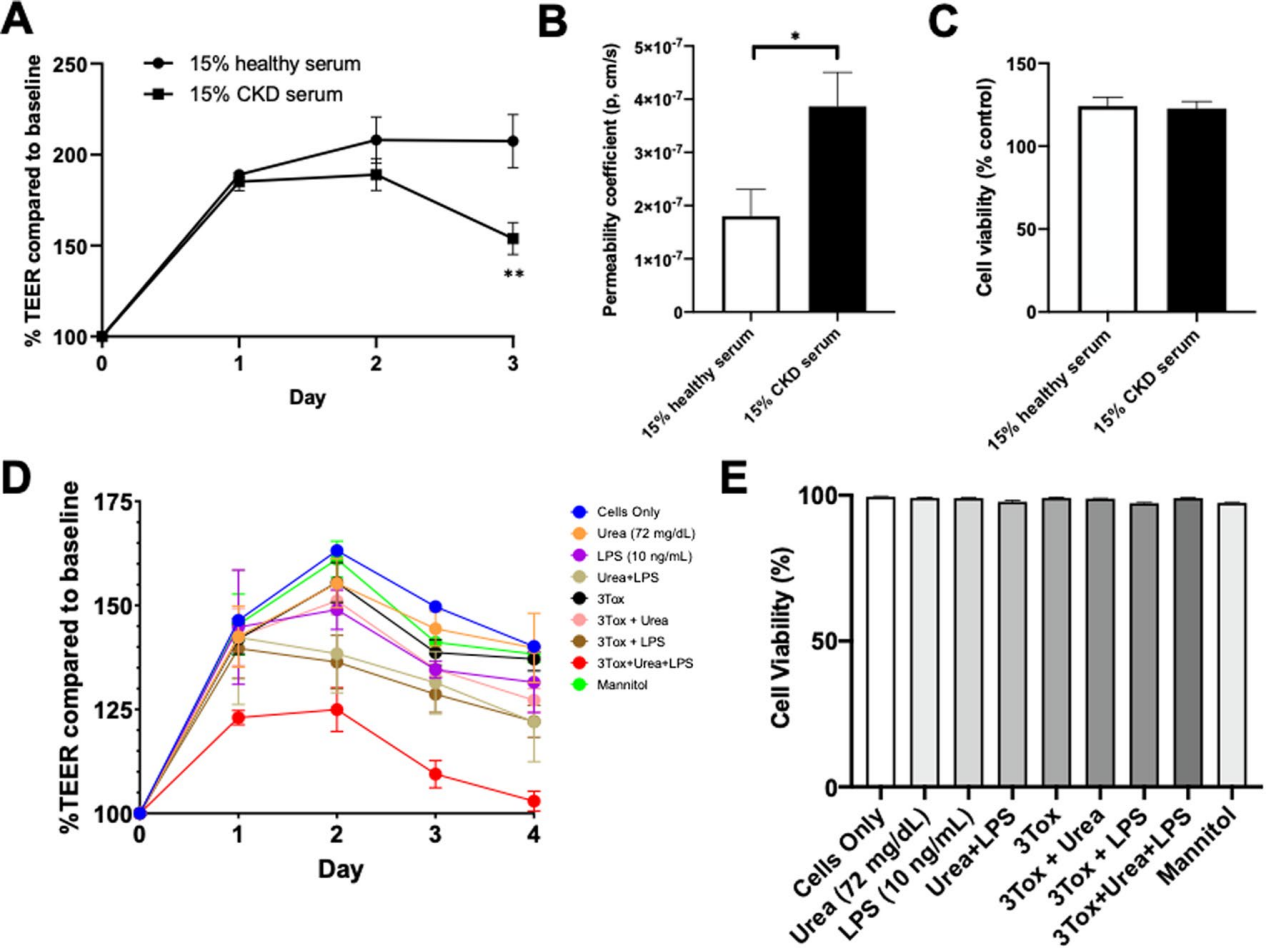
Serum-derived factors in CKD disrupted BBB integrity in vitro. **A**–**C** Effects of CKD dialysis serum on the iPSC-derived human brain microvascular endothelial cells (ihBMEC) monolayer. Incubation with CKD dialysis serum significantly decreased **A** TEER, and increased **B** tracer (sodium fluorescein) permeability across the ihBMEC monolayer 3 days after serum treatment initiation, compared with serum from healthy volunteers. **C** ihBMEC viability measured by CCK8 assay 3 days after serum treatment initiation was not changed. **D**, **E** Effects of uremic toxins on bEnd.3 endothelial barrier. **D** TEER remained significantly lower in the 3Tox + Urea + LPS group compared with the control group. After 4 days of toxin exposure, TEER was significantly lower in the Urea + LPS (p < 0.01), 3Tox + LPS (*p* < 0.05), and 3Tox + Urea + LPS group (*p* < 0.0001) compared with the control group. **E** bEnd.3 cell viability was assessed by trypan blue assay and was equivalent across groups. 3Tox = indoxyl sulfate 150 μg/mL + p-cresyl sulfate 100 μg/mL + trimethylamine N-oxide 1 mg/mL. The reference Cells Only group was cells grown in DMEM with 10% FBS, and mannitol 450 mg/dL was used as an osmotic control group. Data shown are mean ± SEM of at least 3 independent experiments done in duplicate. *p < 0.05 and **p < 0.01

To compare effects of healthy and CKD serum on paracellular passage across the ihBMEC monolayer, ihBMEC were treated with serum from either healthy or dialysis patient donors. TEER values showed some initial increase with both control and CKD serum and plateaued after 2 days of incubation. Following 3 days of incubation, TEER was significantly lower in the CKD serum group (157 ± 8.9% vs. 195 ± 7% in the healthy serum group, relative to baseline TEER values; *p* = 0.01) (Fig. 5A). The passive permeability of the brain endothelial monolayer was also assessed. Permeability coefficients of sodium fluorescein were 1.8 ± 0.5 × 10^–7^ and 3.9 ± 0.6 × 10^–7^ cm/s in cells treated with healthy and CKD serum, respectively, i.e., incubation of ihBMEC with CKD serum doubled the passage of sodium fluorescein (*p* < 0.05) across the monolayer (Fig. 5B). ihBMEC viability, shown as the percentage of control cells without serum treatment, was not affected (Fig. 5C).

### Gut-derived uremic toxins aggravated urea/LPS-induced bEnd.3 endothelial barrier dysfunction

Effects of uremic toxins on the bEnd.3 endothelial barrier were assessed by TEER measurements from day 0 (baseline) to day 4 (Fig. 5D). TEER values were significantly lower in the 3Toxins + Urea + LPS group compared with the control group. After 4 days of incubation, TEER was significantly lower in the Urea + LPS (121 ± 4.74%, *p* < 0.01), 3Toxins + LPS (122 ± 3.83%, *p* < 0.05), and 3Toxins + Urea + LPS group (103 ± 2.38%, *p* < 0.0001) compared with the control group (140 ± 2.48%) (Fig. 5D). bEnd.3 cell viability assessed by trypan blue exclusion was equivalent across groups (Fig. 5E).

## Discussion

In this study, adenine-induced CKD promoted the development of CMH in aged C57BL/6J mice without altering blood pressure levels. CMH number and serum creatinine values were significantly correlated, indicating that the extent of CMH development was directly proportional to degree of renal insufficiency. CKD mice exhibited greater microglial area, a phenotypic switch from resting to activated microglia, and elevated fibrinogen extravasation compared with control mice, indicating CKD-induced microglial activation and BBB disruption in this model. Furthermore, microglial depletion with PLX3397 diet significantly decreased CMH formation in aged CKD mice without affecting serum creatinine levels. We also conducted mechanistic studies using both human and murine in vitro brain microvascular endothelial models. Our results showed that serum-derived factors in CKD disrupted the ihBMEC monolayer by reducing the TEER values of the monolayer and enhancing the passage of sodium fluorescein across the monolayer. Uremic toxins (i.e., IS, PCS, and TMAO) in combination with low-dose urea and LPS induced a marked drop in the TEER of the bEnd.3 cells. Collectively, these findings indicate that CKD provokes microvascular injury, leading to CMH formation in this model.

Investigating the relationship between CKD and CMH development in the clinical setting is challenging due to the presence of comorbidities (e.g., hypertension and diabetes) that can also incur cerebrovascular injury. Elevated blood pressure has been associated with increased CMH burden [21]. In this adenine-induced CKD mouse model, the association of kidney injury with CMH development was independent of blood pressure levels. Therefore, the relationship was examined in a non-hypertensive mouse model of aging and CKD. The increase in the serum biomarker creatinine (Fig. 1B) due to poor renal clearance confirmed impaired kidney function [27], consistent with our previous work with young animals [13]. We previously reported young mice required supplemental injections of LPS to show the full extent of CKD impact on CMH formation [13]. The current study used aged animals, and the impact of CKD was observable without supplemental LPS. This again emphasizes the role of aging in CMH development and may be related to the known heightened microglial reactivity with aging [28]. We showed that CKD enhanced the development of CMH independent of blood pressure, with a greater extent in mice with more severe kidney injury (Fig. 2C). We noted sex difference in CKD-induced CMH development, whereby males showed a more pronounced increase in CMH number compared with female animals (Fig. 2B) in parallel with significantly higher serum creatinine levels (Fig. 1C), and increased microglial activation (Fig. 3B) and fibrinogen extravasation (Fig. 3E) in CKD males than females. These findings suggest that in the CKD model being investigated, aged male mice are more susceptible to adenine-indued kidney injury than the aged females. In accordance with our findings, a number of population-based studies have demonstrated faster CKD progression to end-stage renal disease and higher mortality in men than women [29–34]. While the underlying mechanisms are not completely understood, sex hormones are likely involved in CKD progression by mediating inflammation, oxidative stress, fibrosis, and activation of the renin–angiotensin system [35]. Testosterone exerts a deleterious effect in men by triggering apoptotic pathways leading to nephron cell loss and kidney structural damage [36]. By contrast, estrogen in women is renoprotective, attributable to the beneficial effects on vascular structure and function of endothelial and vascular smooth muscle cells [37]. Female sex hormone effects are less relevant in our study with aged mice; by age 17 months, 80% of female mice have irregular estrous cycles or have ceased cycling [38]. Regardless, our findings have important implications that serum creatinine as a biomarker of kidney dysfunction also serves as a CMH risk marker.

CKD-induced neuroinflammation has been proposed as a key mechanism responsible for neurological dysfunction [39–41]. CKD progression contributed to increased production of pro-inflammatory cytokines and DNA damage in brain cells [40], increasing the likelihood of developing neurological dysfunction in patients with end-stage renal disease. Neuroinflammation is regulated by microglia and astrocytes, the innate immune cells of the central nervous system mediating the inflammatory response in aging, neurodegenerative disease, traumatic brain injury, and ischemic stroke [42–44]. Microglial dynamics are important in maintaining brain homeostasis. Microglia are maintained in a resting state with a morphology characterized by long, ramified processes extending from the soma and terminating with bulbous endings; they can be activated when brain injuries are present, transforming into a phagocytic appearance, exhibiting large, rounded soma with no or few processes [25, 45]. Impaired microglial function has been implicated in aging [46] and Alzheimer’s disease [47]. CKD-induced neuroinflammation has been associated with increased microglia/macrophage recruitment, a shift from an anti-inflammatory M2 to a pro-inflammatory M1 phenotype [48], and the formation of NLRP3 inflammasomes [49]. Although morphological profiles of microglia have been well-characterized in rodents [50], little is known about their morphological changes in the context of CKD, as well as the neuropathological consequences. In the current study, CKD is associated with microglial activation, shown by increased Iba-1 immunoreactivity and a phenotypic switch from resting to activated microglia (Fig. 3A–C).

We used Iba-1 immunostaining which identifies both microglia and macrophages [48]. We characterized the morphology of microglia at higher magnification (40x) to distinguish microglia from macrophages, which allowed us to determine the contribution of microglia in CKD-induced neuroinflammation (Fig. 3F). Our prior mouse work showed a positive correlation between Iba-1 immunoreactivity and CMH number [14]. Thus, the causal relationship between microglial activation and CMH formation was investigated in the current study using PLX3397 diet allowing for microglial depletion (Fig. 4). CKD-induced CMH formation was significantly decreased in aged mice with microglial depletion (Fig. 4F), while serum creatinine levels were found not to be affected (Fig. 4B). These findings indicate that CKD-induced CMH formation is at least partly mediated by microglial activation.

The BBB is formed by endothelial cells lining the capillary wall, astrocyte end-feet surrounding the capillary, and pericytes embedded in the baseline membrane, thus creating a physical barrier between the peripheral circulation and the central nervous system. The tight junctions between the endothelial cells serve to restrict passage of blood-borne substances (e.g., fibrinogen) into the brain and play a crucial role in brain homeostasis [51]. Impaired endothelial tight junctions at the BBB are well-characterized in aging brains [52] and can lead to passage of iron into the brain [53]. We have previously shown disruption in BBB tight junction proteins in young CKD mice during an inflammatory state [13]. Following the previous observations, we examined the passage of fibrinogen into the brains of CKD mice. Fibrinogen enters the brain after BBB injury and can be converted into insoluble fibrin, contributing to neuroinflammation and neuronal damage in many conditions [54–57]. Fibrinogen/fibrin deposition is associated with microglial activation and increased immune cell recruitment into the brain [54, 58]. In line with this finding, we showed microglial activation and increased fibrinogen deposition in the brains of CKD mice (Fig. 3). Activated microglia modulate expression of tight junction proteins essential for BBB integrity [59], which may further exacerbate BBB disruption and CMH formation.

Our assessment of microvascular integrity in vitro has relied on TEER and tracer permeability measurements. TEER is the measurement of electrical resistance across a cellular monolayer to evaluate integrity of the endothelial monolayer. In our in vitro study with ihBMEC, TEER was elevated in the first 24 h due to the trophic factors present in the serum and reached the highest value at 48 h. Incubation with CKD serum caused injury to the monolayer, as shown by a reduction in TEER after serum treatment initiation (Fig. 5A). This is consistent with our previous findings from an in vitro study incubating mouse brain endothelial cells (bEnd.3) with CKD serum [13]. Furthermore, the decrease in TEER was accompanied by a two-fold increase in sodium fluorescein permeability across the ihBMEC monolayer (Fig. 5B). Sodium fluorescein is a small molecular weight tracer (MW: 376 Da) that more readily diffuses through the BBB than larger molecular weight tracers, and therefore has served as a common marker for altered permeability [26]. Previously, we showed an inverse correlation between sodium fluorescein permeability and TEER measurements across the ihBMEC monolayer [26]. Together, impairment of the monolayer induced by CKD-derived serum factors allows for increased passage of sodium fluorescein across the monolayer, suggesting disruption of the endothelial monolayer and consistent with BBB injury observed with fibrinogen immunostaining.

The murine gut microbiome changes with aging and is associated with alterations in microbial carbohydrate metabolism, decreased fecal short-chain fatty acids and decreased cobalamin and biotin biosynthesis [60, 61]. In the current study, we examined well-known gut-derived uremic toxins that are generated in the CKD milieu. These toxins are derived from amino acid catabolism by intestinal microbiota and are associated with systemic inflammation and vascular injury in CKD [62–65]. The exact uremic toxins that contribute to BBB disruption and whether the effects are results of an individual toxin or a combination of several toxins remain largely unknown. We have previously shown CKD serum from dialysis patients caused marked drop in TEER, and urea was one of the key uremic toxins. Exposure of bEnd.3 cells to urea at concentrations approximate to the values measured in dialysis patients reduced TEER in a dose-dependent manner [13]. In the current in vitro study, this was further investigated by exposing bEnd.3 mouse brain endothelial cells with various gut-derived uremic toxins alone and in combination, with TEER measurements every 24 h. We demonstrated that uremic toxins (i.e., IS, PCS, and TMAO) in combination with urea and LPS exerted the most deleterious effects on the endothelial barrier. TEER was significantly lower in the 3Toxins + Urea + LPS group compared with the control group, and eventually declined to a level close to the baseline values at day 4 (Fig. 5D). These findings suggest gut-derived uremic toxins aggravate urea/LPS-induced bEnd.3 endothelial barrier dysfunction, again consistent with findings of BBB injury observed with fibrinogen immunostaining.

Despite these multiple findings, this study has some limitations. The mouse study suggests a key role of microglia in mediating CMH formation in CKD animals. It should be noted that the use of CSF1R inhibitor PLX3397 eliminates microglia in the parenchyma, but also affects the number of non-parenchymal macrophages in the perivascular spaces, the choroid plexus, and the meninges [66], which are known to cause neurovascular dysfunction [67]. Our findings do not rule-out a contribution of perivascular macrophages to CMH formation [68], a subject that warrants further investigation. The binary morphological categorization of microglia into resting and activated states may be an oversimplification. Microglia intermediate between the two states may adopt various functions in immune cell recruitment and activation, cell proliferation, and phagocytosis as their morphology changes [69]. Note that the vascular source of CMH in uremic milieu remains unclear, as iron uptake into the brain is highly regulated by BBB. Mouse models and human postmortem studies of aging, hypertension, and Alzheimer’s disease have suggested a capillary source of CMH involving BBB disruption [14, 70–72], consistent with the findings from the in vitro CKD models we investigated. However, these findings should be interpreted with caution, because CMH may develop via a mechanism independent of capillary injury [73–75]. To expand our knowledge on the vascular source of CMH, we have developed a semi-automated approach to characterize microvascular network in three-dimensional (3D) imaging of mouse brains [76]; this will enable us to visualize the co-localization of fluorescently labeled microvascular network and Prussian blue-positive CMH and therefore, identify the vascular origin of CMH. In addition, the issue of cognitive decline with CKD is important, as we have emphasized [77]. However, the experiments in our manuscript were neither designed nor powered to address this issue. In terms of in vitro BBB models, recent studies show that ihBMEC have a mixed endothelial–epithelial transcriptional profile [26, 78, 79], and we therefore used two different brain endothelial cell culture systems, i.e., ihBMEC and bEnd.3 cells, for a better understanding of the mechanistic role of microvascular endothelial function in CKD-induced CMH formation.

## Conclusions

Adenine-induced CKD promotes the development of CMH in aged C57BL/6J mice independent of blood pressure, likely via microglial activation and BBB disruption. Extent of CMH development is directly proportional to degree of renal insufficiency. Moreover, serum-derived factors in CKD disrupt endothelial monolayer by reducing TEER and enhancing the passage of sodium fluorescein across the monolayer. Gut-derived uremic toxins (i.e., IS, PCS, and TMAO) aggravated urea/LPS-induced endothelial barrier dysfunction by producing a marked drop in TEER, supporting the key role of uremic toxins in CKD-specific mechanisms that contribute to microvascular dysfunction. These findings indicate that CKD provokes microvascular injury leading to CMH formation in this model and suggest that CKD makes an important contribution to cerebral small vessel disease.

## Data Availability

Data are available upon reasonable request.

## Abbreviations

ABC: Avidin–biotin-peroxidase
BBB: Blood–brain barrier
bFGF: Basic fibroblast growth factor
CCK-8: Cell Counting Kit-8
CKD: Chronic kidney disease
CMH: Cerebral microhemorrhages
CSF1R: Colony-stimulating factor 1 receptor
DAB: 3,3′-Diaminobenzidine
DMEM: Dulbecco’s Modified Eagle’s Medium
DMEM/Ham’s F12: Dulbecco's Modified Eagle Medium/Ham’s nutrient mixture F-12
FBS: Fetal bovine serum
GFAP: Glial fibrillary acidic protein
hESFM: Human endothelial serum-free medium
ihBMEC: Human brain microvascular endothelial cells
IMR90-4: IMR90 clone 4 line
iPSC: Human induced pluripotent stem cell
IS: Indoxyl sulfate
LC–MS/MS: Liquid chromatography with tandem mass spectrometry
LDL: Low-density lipoprotein
LPS: Lipopolysaccharide
MEM–NEAA: Minimum essential medium–nonessential amino acids
PBS: Phosphate-buffered saline
PCS: P-cresyl sulfate
ROCK: Rho-associated protein kinase
RT: Room temperature
TEER: Transendothelial electrical resistance
TMAO: Trimethylamine-N-oxide
vWF: Von Willebrand factor
